# Loss of Pinch Proteins Causes Severe Degenerative Disc Disease-Like Lesions in Mice

**DOI:** 10.14336/AD.2023.0212

**Published:** 2023-10-01

**Authors:** Xiaohao Wu, Mingjue Chen, Sixiong Lin, Sheng Chen, Jingliang Gu, Yuchen Wu, Minghao Qu, Weiyuan Gong, Qing Yao, Huiping Li, Xuenong Zou, Di Chen, Guozhi Xiao

**Affiliations:** ^1^Department of Biochemistry, School of Medicine, Shenzhen Key Laboratory of Cell Microenvironment, Guangdong Provincial Key Laboratory of Cell Microenvironment and Disease Research, Southern University of Science and Technology, Shenzhen, China.; ^2^Guangdong Provincial Key Laboratory of Orthopaedics and Traumatology, Department of Spine Surgery, The First Affiliated Hospital of Sun Yat-sen University, Guangzhou, China.; ^3^Department of Orthopedics, Union Hospital, Tongji Medical College, Huazhong University of Science and Technology, Wuhan 430022, Hubei, China.; ^4^Department of Orthopedics, Shanghai municipal Hospital of Traditional Chinese Medicine, Shanghai University of Traditional Chinese Medicine, China.; ^5^Department of Endocrinology, Chongqing Traditional Chinese Medicine Hospital, Chongqing, China.; ^6^Department of Respiratory and Critical Care Medicine, Shenzhen People’s Hospital, Southern University of Science and Technology, Shenzhen, China.; ^7^Research Center for Human Tissues and Organs Degeneration, Shenzhen Institutes of Advanced Technology, Chinese Academy of Sciences, Shenzhen, China.

**Keywords:** Pinch, disc degeneration, inflammation, anti-TNFα therapy, Adalimumab

## Abstract

Degenerative disc disease (DDD) is one of the most common skeletal disorders affecting aged populations. DDD is the leading cause of low back/neck pain, resulting in disability and huge socioeconomic burdens. However, the molecular mechanisms underlying DDD initiation and progression remain poorly understood. Pinch1 and Pinch2 are LIM-domain-containing proteins with crucial functions in mediating multiple fundamental biological processes, such as focal adhesion, cytoskeletal organization, cell proliferation, migration, and survival. In this study, we found that Pinch1 and Pinch2 were both highly expressed in healthy intervertebral discs (IVDs) and dramatically downregulated in degenerative IVDs in mice. Deleting Pinch1 in aggrecan-expressing cells and Pinch2 globally (*Aggrecan^CreERT2^; Pinch1^fl/fl^; Pinch2^-/-^*) caused striking spontaneous DDD-like lesions in lumbar IVDs in mice. Pinch loss inhibited cell proliferation and promoted extracellular matrix (ECM) degradation and apoptosis in lumbar IVDs. Pinch loss markedly enhanced the production of pro-inflammatory cytokines, especially TNFα, in lumbar IVDs and exacerbated instability-induced DDD defects in mice. Pharmacological inhibition of TNFα signaling mitigated the DDD-like lesions caused by Pinch loss. In human degenerative NP samples, reduced expression of Pinch proteins was correlated with severe DDD progression and a markedly upregulated expression of TNFα. Collectively, we demonstrate the crucial role of Pinch proteins in maintaining IVD homeostasis and define a potential therapeutic target for DDD.

## INTRODUCTION

The intervertebral disc (IVD) is a unique weight-bearing structure located between the vertebral bodies of the spine. The main structure of IVDs includes annulus fibrosus (AF), nucleus pulposus (NP), and cartilaginous plates (CEP) [1]. During the last decade, degenerative disc disease (DDD) has become one of the most common skeletal disorders with a rapidly increasing prevalence worldwide, especially in aged populations [2]. The progression of DDD leads to clinical symptoms, such as low back/neck pain, resulting in disability and huge socioeconomic burdens [3]. Currently, the management and treatment of DDD can only relieve the symptoms, which cannot provide fundamental and long-term therapeutic benefits [4, 5]. This is in part due to an incomplete understanding of the DDD pathogenesis [5]. Thus, it is highly desirable to investigate the pathological mechanisms underlying DDD onset and progression, which will help to develop new therapeutic approaches for the prevention and treatment of this devastating disease.

Focal adhesions (FAs) are macromolecular assemblies that connect the cell cytoskeleton to the extracellular matrix (ECM) for controlling multiple fundamental cellular processes, including FA dynamics, cell-ECM adhesion and migration, proliferation, survival, mechanosensation, and signal transduction [6-22]. The Pinch proteins are a family of LIM domain-containing proteins that function as key components of the FA complexes [23, 24]. In mammals, there are two functional Pinch proteins, i.e., Pinch1 and Pinch2 [24]. Pinch1 global-deficient (*Pinch1-/-*) mice are embryonically lethal [25], whereas Pinch2-null (*Pinch2^-/-^*) mice display no apparent phenotypes [26]. Previous studies have mainly focused on the crucial functions of Pinch proteins in the malignancies [27]. A high expression of Pinch1 has been found in human laryngeal carcinoma samples and is correlated with a poor prognosis. Moreover, it has been reported that Pinch1 promotes tumor growth by regulating mitochondrial dynamics, proline synthesis, and cell proliferation [28]. Emerging evidence revealed that Pinch proteins regulate organ formation and homeostasis [24, 25, 29-32]. For instance, Liang and coworkers have reported an indispensable role of Pinch proteins in heart morphogenesis and function [31]. We have demonstrated that Pinch proteins control bone, cartilage, and adipose tissue development and homeostasis through distinct molecular mechanisms [29, 30, 32]. However, whether Pinch proteins are involved in IVD homeostasis and disease remains unknown.

In this study, we found that Pinch1 and Pinch2 were both highly expressed in cells of AF, NP, and CEP in healthy adult IVDs and dramatically downregulated in aging- and lumbar spine instability (LSI)-induced DDD in mice. By generating a series of genetic mouse models, we investigated the effects of Pinch1 deletion in aggrecan-expressing IVD cells using *Aggrecan^CreERT2^* mice with and without global Pinch2 inactivation in mice. We found that Pinch1 and Pinch2 functionally compensated for each other, and the loss of both factors resulted in multiple striking spontaneous DDD-like lesions and enhanced progression of LSI-induced disc defects in mice.

## MATERIALS AND METHODS

### Human NP samples

Human NP samples were collected from 12 DDD patients who underwent nucleotomy surgery. The demographic information of enrolled DDD patients is shown in Supplementary Table 1. The degenerative grade of the NP specimens was classified using an MRI-based Pfirrmann grading system. NP specimens with grade II-III defects were considered mild DDD, whereas NP specimens with grade IV-V defects were considered severe DDD. Ethics approval was obtained from the Ethics Committee of Tongji Medical College, Huazhong University of Science and Technology (No. [2021] IEC (134)). Informed consent was obtained from each participant enrolled in this study.

### Animal Studies

The generation of floxed Pinch1 (*Pinch1^fl/fl^*) and Pinch2 global knockout (*Pinch2^-/-^*) mice was previously described [29-33]. The genotyping primer information is listed in Supplementary Table 2. *Pinch1^fl/fl^* mice were bred with the *Aggrecan^CreERT2^* knock-in transgenic mice to obtain *Aggrecan^CreERT2^; Pinch1^fl/fl^* mice. To generate the Pinch1/2 double knockout (dKO) mice, the *Aggrecan^CreERT2^; Pinch1^fl/fl^* mice were first crossed with *Pinch2^-/-^* mice to generate *Aggrecan^CreERT2^; Pinch1^fl/+^; Pinch2^+/-^* mice. Then, the *Aggrecan^CreERT2^; Pinch1^fl/+^; Pinch2^+/-^* mice were crossed with each other and generate the *Aggrecan^CreERT2^; Pinch1^fl/fl^; Pinch2^-/-^* mice. At 2 months of age, the *Aggrecan^CreERT2^; Pinch1^fl/fl^* or *Aggrecan^CreERT2^; Pinch1^fl/fl^; Pinch2^-/-^* mice were subjected to five continuous injections of tamoxifen (TAM) (100 mg/kg body weight per day) to induce conditional deletion of the *Pinch1* gene in aggrecan-expressing IVD cells. Age-matched male mice were treated with corn oil and used as controls. For induction of instability-induced DDD, LSI surgery was performed in L3-L5 lumbar IVDs of 2-month-old male mice as previously described [34]. To test whether pharmacological inhibition of TNFα attenuates the DDD-like lesions caused by Pinch loss, 2-month-old male *Aggrecan^CreERT2^; Pinch1^fl/fl^; Pinch2^-/-^* mice were injected with TAM/corn oil and, one week later, subcutaneously injected with PBS or Adalimumab (1mg/kg body weight every 2 days) for another 2 months. Male mice were used in this study for consistency and to minimize the use of animals (*N* = 6-7 per group). All mice were housed at 22 ± 2- and exposed to 12 hours/12 hours of light-dark cycles. All animal experiments were approved by the Institutional Animal Care and Use Committee of the Southern University of Science and Technology (No. SUSTC-JY2020119).

### Histology

The decalcification, dehydration, and paraffin embedding of lumbar IVDs samples were performed according to our previously established protocols [10]. The paraffin-embedded IVD samples were cut into 5-µm thick sections and stained with safranin O & fast green (SO&FG) (Solarbio, Cat#G1371) as previously described [10, 16, 19]. The severity of DDD-like lesions was evaluated using a semi-quantitative histological grading system in a double-blinded manner [35]. All histological scores were assessed by three independent scorers, and the average values were calculated and presented. All representative images were selected based on the mean values of histological scores. Human NP samples were fixed in 4% paraformaldehyde (PFA) for 48 hrs and then dehydrated, paraffin-embedded, and cut into 5-μm sections. Alcian blue and hematoxylin & eosin (H&E) staining were performed to determine the degenerative degree of human NP samples as previously described [9, 10, 20].

### Quantitative immunofluorescent analyses

For immunofluorescent (IF) staining, 5-µm lumbar IVD sections were hydrated and permeabilized with Immunostaining Permeabilization Solution with Saponin (Beyotime, Cat# P0095) for 5 mins at room temperature (RT), blocked with Immunol Staining Blocking Buffer (Beyotime, Cat# P0102) for 1h at RT, and then incubated with primary antibodies (Supplementary Table 3) overnight at 4°C. After washing in PBS with 0.1% Tween 20, the sections were incubated with Goat anti-Rabbit IgG (H+L) Cross-Adsorbed Secondary Antibody, Alexa Fluor 488 (Invitrogen, Cat# A-11008) (1:400) for 1h at RT. Isotype antibody (Normal Rabbit IgG, Sigma, NI01) controls and secondary antibody-only controls were employed to validate antibody specificity and distinguish genuine target staining from the background. The fluorescent signals in IVDs were determined using a Leica SP8 Confocal Microsystems and analyzed using Image J (version 1.53k) software as previously described ^[10, 36]^. Representative images were selected based on the mean values of fluorescent signals.

### TUNEL staining

Cell apoptosis was evaluated using the One Step TUNEL Apoptosis Assay Kit (Red Fluorescence) (Beyotime, C1090) as previously described [12, 16, 29].

### Cell culture and siRNA knockdown experiments

The immortalized rat NP cell line was described in previous studies [37]. The NP cells were cultured in DMEM supplemented with 10% fetal bovine serum (Gibco; 10099-141) and 1% penicillin and streptomycin (Hyclone; SV30010). For in vitro knockdown of Pinch1/2 expression, the NP cells were transfected with Pinch1/2 siRNA using a Lipofectamine RNAiMAX transfection reagent (Invitrogen, Cat# 13778075) as previously described [10, 19, 38, 39]. NP cells transfected with negative control siRNA were used as the control group. Cell attachment assay was performed according to our previously established protocol [19]. The siRNA sequences used in this study are shown in Supplementary Table 4. A customized compression apparatus (ZL 201120082425.3) was utilized for the CL experiment following our previously established protocol [10]. The apparatus provides 1 MPa CL to mimic the abnormal mechanical loading condition in IVDs.

### Statistical Analysis

The sample size for each experiment was determined based on our previous experience. All mice used in this study were randomly assigned to each group. Statistical analyses were completed using the Prism GraphPad. Results were expressed as mean ± standard deviation (s.d.). The normality of data was tested for all variables using the Kolmogorov-Smirnov (K-S) test. For normally distributed data, a two-tailed unpaired Student’s *t* test was used to determine the statistical difference between the two groups. For non-normally distributed data, an unpaired nonparametric Mann-Whitney test was used to determine the statistical difference between the two groups. A two-way ANOVA test was used to determine the statistical difference between groups with two independent variables. Differences with *P*<0.05 were considered statistically significant.

## RESULTS

### Downregulated Pinch expression in degenerative IVDs in mice

**Figure 1.**
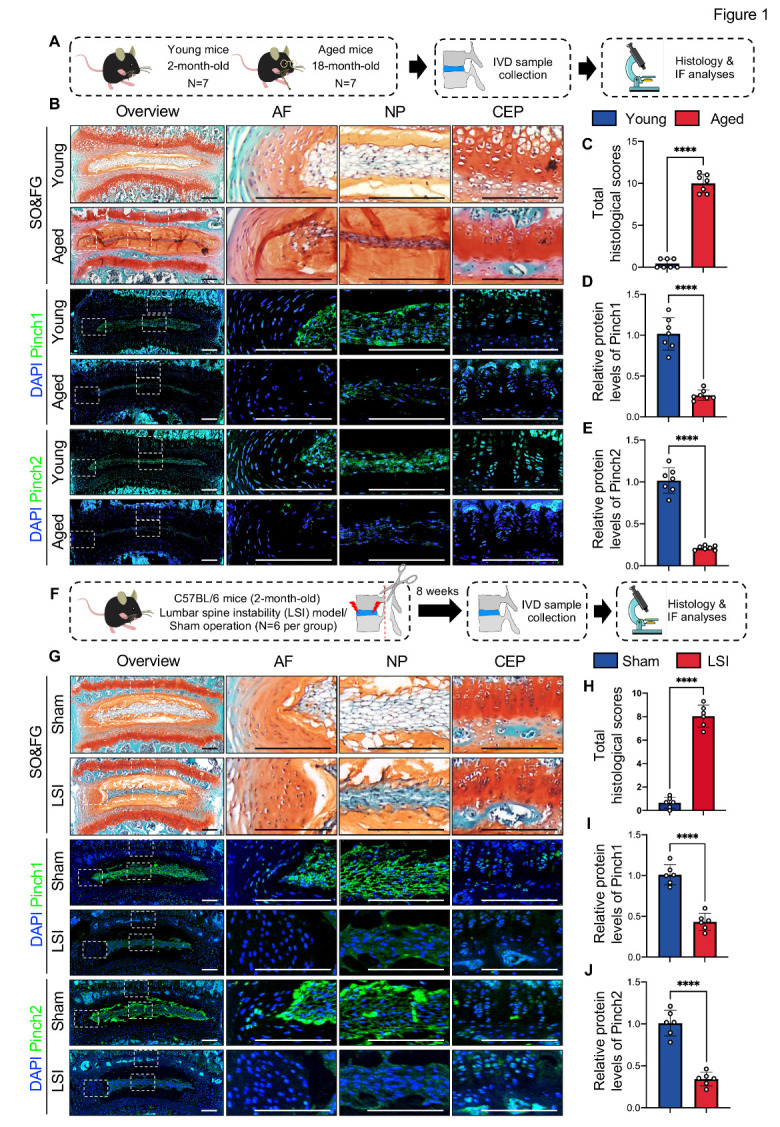
Pinch proteins were highly expressed in healthy adult IVDs and downregulated in degenerative IVDs in mice. (A) A schematic diagram illustrating the experimental design. *N = 7* per group. (B) Representative images of SO&FG and IF staining of lumbar IVD sections from young (2-month-old) and aged (18-month-old) mice. White dashed boxes indicate the higher magnification images of AF, NP, and CEP in the right panels. Scale bar: 200 µm. (C) Total histological scores of lumbar IVDs. (D, E) Relative protein expressions of Pinch1 and Pinch2 in lumbar IVDs normalized to the mean value of the young group. (F) Overview of the LSI experiment. (G) Representative images of SO&FG and IF staining of lumbar IVD sections from mice at 8 weeks after LSI surgery or sham operation. White dashed boxes indicate the higher magnification images of AF, NP, and CEP in the right panels. Scale bar: 200 µm. (H) The total histological scores of lumbar IVDs. (I, J) Relative protein expressions of Pinch1 and Pinch2 in lumbar IVDs normalized to the mean value of the sham group. Results are expressed as mean ± standard deviation (s.d.). ****P < 0.0001. In C-E and H-J, a two-tailed unpaired Student’s *t* test was used for statistical analyses.

As an initial step to investigate whether Pinch proteins play a role in aging-related DDD pathogenesis, we collected lumbar IVD samples from young (2-month-old) and aged (18-month-old) mice and performed SO&FG and IF staining as indicated in Figure 1A. SO&FG staining showed that, in young IVDs, the NP compartment was well-defined and rich in vacuolar NP cells (Fig. 1B). In contrast, aged IVDs displayed several DDD-like pathological changes, including decreased cellularity, loss of clear boundary between NP and AF, NP fibrosis, and abnormal ossification of CEP (Fig. 1B). Using a histological scoring system [35], we evaluated the severity of these DDD-like defects in both groups. The result showed that the total histological scores of aged IVDs were significantly higher than those of young IVDs (Fig. 1C). Furthermore, IF staining revealed that Pinch1 and Pinch2 proteins were both highly expressed in cells of AF, NP, and CEP in young IVDs, which were drastically decreased in aged IVDs (Fig. 1B, D, E). Next, we investigated whether Pinch1/2 expression is altered in the LSI-induced DDD model in mice. We performed LSI surgery in L3-L5 IVDs of 2-month-old male C57BL/6 mice to induce DDD-like damage (Fig. 1F). Age- and sex-matched mice with sham operations served as controls. All mice were sacrificed eight weeks after LSI surgery, and the lumbar IVDs were collected. SO&FG staining confirmed the onset of DDD in lumbar IVDs of LSI-treated mice (Fig. 1G, H). Similar to results from aged mice, IF staining indicated that the protein expression of Pinch1/2 was diminished in LSI-treated IVDs compared to sham-operated IVDs (Fig. 1G, I, J).

### Deleting Pinch expression caused striking spontaneous DDD-like defects in mice

Based on the above observations, we investigated whether Pinch1 or Pinch2 expression is essential for maintaining IVD homeostasis in mice. To this end, we generated *Aggrecan^CreERT2^; Pinch1^fl/fl^* mice by crossing *Pinch1^fl/fl^* mice with *Aggrecan^CreERT2^* transgenic mice as indicated in Supplementary Fig. 1A. At 2 months of age, the *Aggrecan^CreERT2^; Pinch1^fl/fl^* male mice were subjected to five continuous injections of TAM (100 mg/kg body weight per day) to induce conditional deletion of the *Pinch1* gene in aggrecan-expressing IVD cells (hereafter referred to as P1cKO). Of note, age-matched male *Aggrecan^CreERT2^; Pinch1^fl/fl^* mice treated with corn oil were used as controls. All mice were sacrificed six months after TAM injections, and IVD samples were harvested. Histological analyses revealed no significant difference in IVD structure between control and P1cKO IVDs (Supplementary Fig. 1B, C). Consistent with a previous report that *Aggrecan^CreERT2^* is active in all IVD tissues, including AF, NP, and CEP [40], the protein expression of Pinch1 was dramatically decreased in cells of those tissues in P1cKO mice (Supplementary Fig. 1B, D). Interestingly, the expression level of Pinch2 protein was markedly upregulated in IVDs of P1cKO mice compared to those of control mice (Supplementary Fig. 1B, E). To determine whether Pinch2 loss affects IVD structure and Pinch1 expression, we collected lumbar IVD samples from 8-month-old *Pinch2^-/-^* (hereafter referred to as P2KO) mice (Supplementary Fig. 2A). Compared with control (*Pinch2^+/+^*) mice, P2KO mice displayed no marked alterations in IVD structure (Supplementary Fig. 2B, C). In addition, IF staining analyses showed that deletion of Pinch2 caused a significant increase in Pinch1 expression in lumbar IVDs of P2KO mice relative to control mice (Supplementary Fig. 2B, D, E).

**Figure 2.**
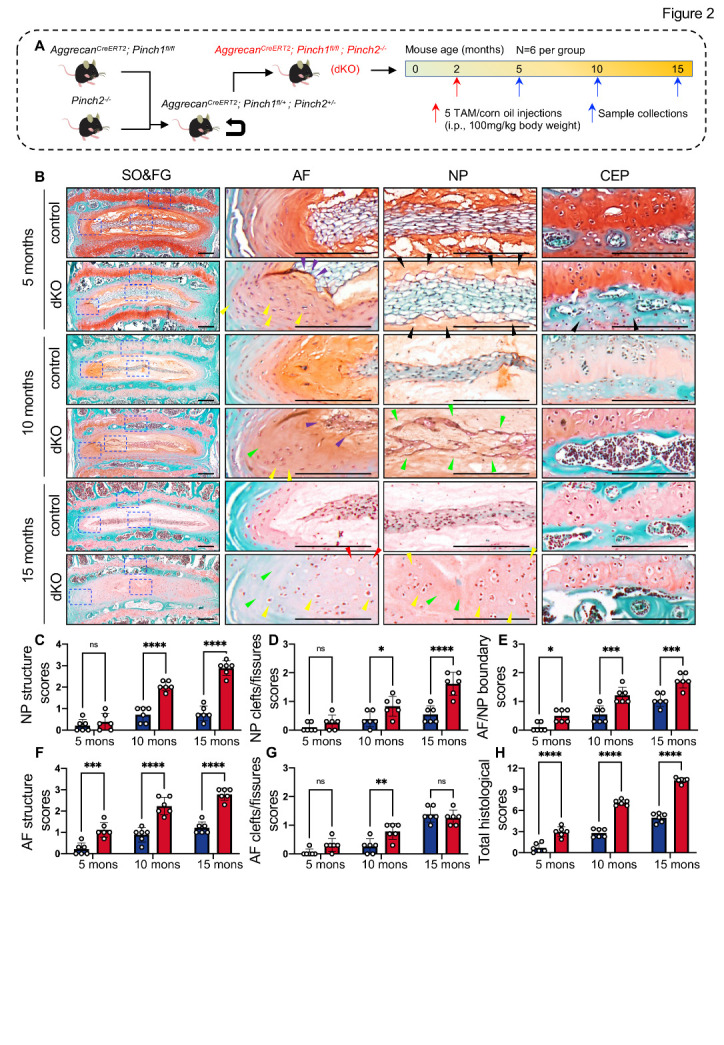
Double knockout of Pinch1 and Pinch2 resulted in spontaneous DDD-like lesions in mice. (A) A schematic diagram illustrating the experimental design.*N = 6* per group. (B) Representative images of SO&FG-stained lumbar IVD sections from control and dKO mice at 5, 10, and 15 months of age. Blue dashed boxes indicate the higher magnification images of AF, NP, and CEP in the right panels. Purple arrows indicate the reversal of AF lamellae into the NP compartment; Yellow arrows indicate the appearance of numerous disorganized chondrocyte-like cells; Black arrows indicate the loss of safranin O-staining; Green arrows indicate the decreased cellularity in IVDs; Red arrows indicate the complete loss of boundary between AF and NP; Scale bar: 200 µm. (C-H) The severity of DDD-like phenotypes was analyzed by grading histological sections using the NP structure scores (C), NP clefts/fissures scores (D), AF/NP boundary scores (E), AF structure score (F), and AF clefts/fissures scores (G). The total histological scores are summed in (H). Results are expressed as mean ± standard deviation (s.d.). *P < 0.05; **P < 0.01; ***P < 0.001; ****P < 0.0001; ns: not significant. In C-H, a two-way ANOVA test was used for statistical analyses.

The above results suggest that Pinch1 and Pinch2 may functionally compensate for each other in IVDs. To determine if this was the case, we crossed the *Aggrecan^CreERT2^; Pinch1^fl/fl^* mice with the *Pinch2^-/-^* mice to produce *Aggrecan^CreERT2^; Pinch1^fl/fl^; Pinch2^-/-^* mice (Fig. 2A and Supplementary Fig. 3). Two-month-old male *Aggrecan^CreERT2^; Pinch1^fl/fl^; Pinch2^-/-^* mice were treated with TAM (hereafter referred to as dKO) or corn oil (control group), as indicated in Figure 2A. At 5, 10, and 15 months of age, mice were sacrificed, and lumbar IVDs were collected (*N = 6* per group for each time point). IF staining confirmed that the expression of both Pinch1 and Pinch2 proteins was essentially abolished in cells of dKO IVDs (Supplementary Fig. 4A-C). Histological analyses showed that dKO mice displayed early features of DDD in lumbar IVDs as early as 3 months after TAM injections, including reversal of AF lamellae into the NP compartment (Fig. 2B, purple arrows), the appearance of numerous disorganized chondrocyte-like cells (Fig. 2B, yellow arrows), and loss of safranin O-stained proteoglycan in NP and CEP (Fig. 2B, black arrows). Starting from 8 months post-TAM injections, severe DDD-like lesions, including markedly decreased cellularity (Fig. 2B, green arrows) and complete loss of boundary between AF and NP (Fig. 2B, red arrows), were observed in dKO mice. Quantitative analyses revealed significantly higher histological scores, including NP structure scores (Fig. 2C), NP clefts/fissures scores (Fig. 2D), AF/NP boundary scores (Fig. 2E), AF structure scores (Fig. 2F), AF clefts/fissures scores (Fig. 2G), and total histological scores (Fig. 2H), in dKO IVDs when compared to those in control IVDs.

### Pinch loss promoted ECM degradation in IVDs and impaired NP cell-ECM adhesion

**Figure 3.**
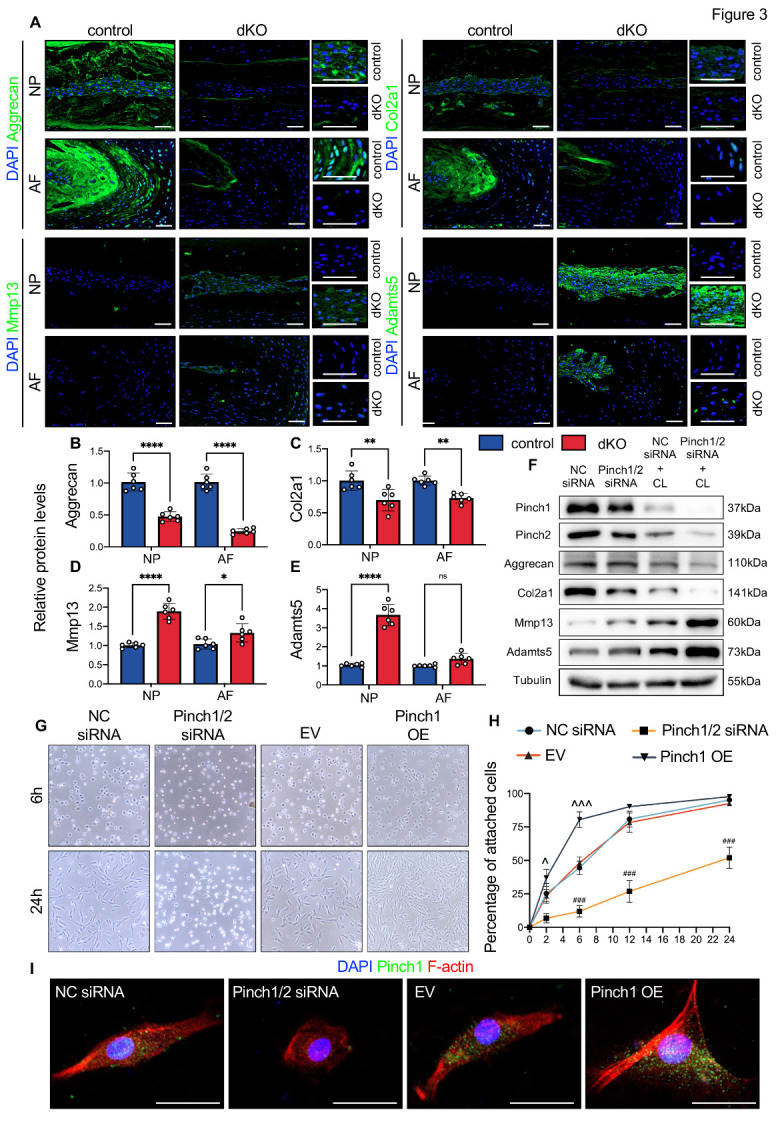
Pinch loss induced ECM degradation and impaired the attachment and spreading of NP cells. (A) Representative IF staining for expression of aggrecan, Col2a1, Mmp13, and Adamts5 using lumbar IVD sections from control or dKO mice at 10 months of age. Higher-magnification images are shown on the right panels. Scale bar: 200 µm. (B-E) Quantitative analyses of the relative protein levels of aggrecan, Col2a1, Mmp13, and Adamts5 in NP or AF using IF-stained sections. The relative protein levels were calculated by normalizing each measured value to the mean value of the control group. *N = 6* mice per group. (F) Western blotting analyses of Pinch1, Pinch2, aggrecan, Col2a1, Mmp13, and Adamts5 in NP cells transfected with negative control siRNA (NC siRNA) or Pinch1/2 siRNA for 24 hours with or without CL treatment. Tubulin was used as an endogenous control. Experiments were repeated three times independently with similar results. (G) Representative images of attachment and spreading of NP cells on type II collagen-coated surfaces after transfection of NC siRNA, Pinch1/2 siRNA, empty vector (EV), and Pinch1 expressing vector (Pinch1 OE), respectively. (H) Percentages of attached cells. (I) Representative IF staining of Pinch1 (green) and F-actin (red) in NP cells transfected with NC siRNA, Pinch1/2 siRNA, EV, and Pinch1 OE, respectively. Nuclei were stained by DAPI. Scale bar: 25 µm. Results are expressed as mean ± standard deviation (s.d.). *P < 0.05; **P < 0.01; ****P < 0.0001; ns: not significant. ^###^*P* < 0.001 vs NC siRNA group; ^*P* < 0.05 vs EV group; ^^^*P* < 0.001 vs EV group. In B-E and H, a two-way ANOVA test was used for statistical analyses.

IF staining revealed that the expression of anabolic ECM proteins (i.e., aggrecan and Col2a1) was downregulated, and that of ECM-degrading enzymes Mmp13 and Adamts5 was upregulated in AF and NP of lumbar IVDs in dKO mice (Fig. 3A-E). We further knocked down Pinch expression by siRNAs in NP cells and determined their effects on ECM metabolism under normal conditions and abnormal compression loading (CL). Western blotting analyses revealed that CL drastically reduced the expression of Pinch1, Pinch2, aggrecan, and Col2a1 proteins and increased that of Adamts5 and Mmp13 in NP cells (Fig. 3F and Supplementary Fig. 5). Knockdown of Pinch by siRNAs significantly reduced Col2a1 expression under normal conditions and exacerbated the molecular changes induced by CL (Fig. 3F and Supplementary Fig. 5). The adhesion and spreading of NP cells on collagen-II-coated surfaces were significantly impaired by Pinch loss but accelerated by Pinch1 overexpression (Fig. 3G-I). Furthermore, Pinch loss significantly decreased the protein expression of total and phosphorylated Smad2/3 and upregulated that of phosphorylated p65 in NP cells both in vivo and in vitro (Supplementary Fig. 6).

### Pinch loss inhibited cell proliferation and induced apoptosis in lumbar IVDs in mice

We further assessed whether Pinch loss affects the proliferative activity of IVD cells by performing IF staining of cell proliferation marker Ki67. In control mice, Ki67 was strongly detected in cells of AF and NP in lumbar IVDs (Supplementary Fig. 7A). However, the protein expression levels of Ki67 decreased by 39% and 57% in NP and AF, respectively, in lumbar IVDs of dKO mice compared with those in control mice (Supplementary Fig. 7B). Results from the terminal deoxynucleotidyl transferase-mediated nick-end labeling (TUNEL) staining revealed increased cell apoptosis in lumbar IVDs of dKO versus control mice (Supplementary Fig. 8A). Quantitative fluorescent analyses revealed that the percentages of TUNEL-positive cells were increased by 14.2- and 5.3-fold in AF and NP, respectively, in lumbar IVDs of dKO mice compared to those in control mice (Supplementary Fig. 8B). In addition, the expression levels of pro-apoptotic proteins (i.e., active Caspase 3 and Bax) were significantly upregulated, whereas the level of anti-apoptotic protein Bcl2 was downregulated in cells of AF and NP tissues of dKO versus control IVDs (Supplementary Fig. 8A, C-E).

### Pinch loss exacerbated LSI-induced DDD lesions in mice

**Figure 4.**
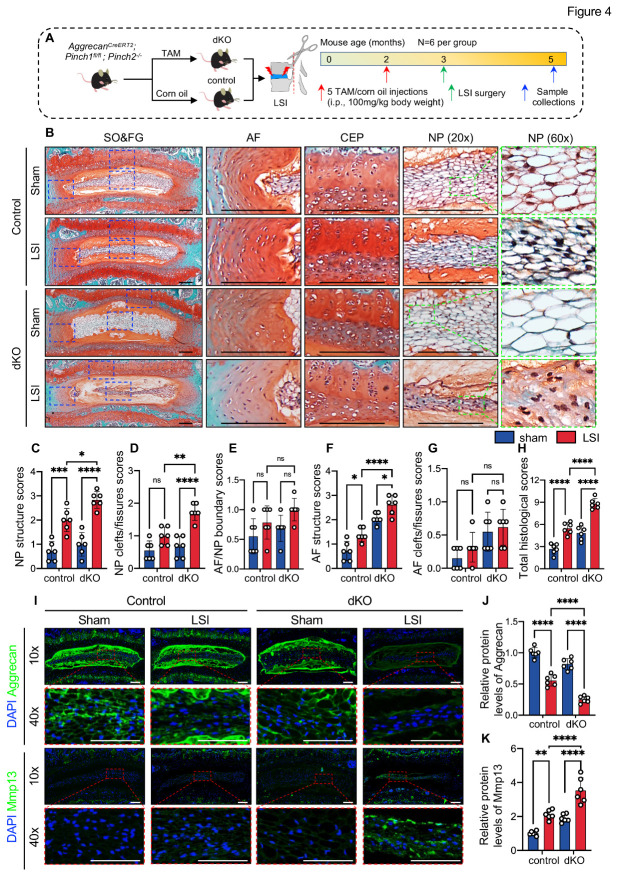
Pinch loss exacerbated LSI-induced disc lesions in mice. (A) A schematic diagram illustrating the experimental design. (B) Representative images of SO&FG-stained lumbar IVD sections from control and dKO mice at 2 months after LSI surgery. Blue dashed boxes indicate the higher magnification images of AF, NP, and CEP in the right panels. Scale bar: 200 µm. (C-H) Quantifications of histological scores. *N* = 6 per group. (I) IF staining of aggrecan and Mmp13 in lumbar IVDs of control and dKO mice. The higher magnification images of NP (red dashed boxes) are shown in the lower panels. Scale bar: 200 µm. (J, K) Relative protein levels of aggrecan and Mmp13 in NP cells normalized to the mean value of the sham-control group. *N = 6* per group. Results are expressed as mean ± standard deviation (s.d.). *P < 0.05; **P < 0.01; ***P < 0.001; ****P < 0.0001; ns: not significant. In C-H and J-K, a two-way ANOVA test was used for statistical analyses.

We next determined whether Pinch loss impacts the progression of LSI-induced DDD in mice. At 2 months of age, the *Aggrecan^CreERT2^; Pinch1^fl/fl^; Pinch2^-/-^* mice were injected with TAM or corn oil. One week later, mice were subjected to sham or LSI surgery, as indicated in Figure 4A. All mice were killed at twelve weeks after LSI surgery, and lumbar IVDs were collected. As expected, LSI-treated control mice exhibited apparent DDD-like defects (Fig. 4B-H). LSI-treated dKO mice displayed dramatic increases in DDD-related pathological parameters, including NP structure scores (Fig. 4C), NP clefts/fissures scores (Fig. 4D), AF structure scores (Fig. 4F), when compared with those in LSI-treated control mice. Notably, the total histological scores were increased by 1.60-fold in LSI-treated dKO IVDs compared to those in LSI-treated control IVDs (Fig. 4H). In addition, Pinch loss significantly aggravated the ECM degradation caused by LSI, as revealed by IF staining of Aggrecan and Mmp13 (Fig. 4I-K).

### Blocking of TNFα signaling mitigated the DDD-like lesions caused by Pinch loss

**Figure 5.**
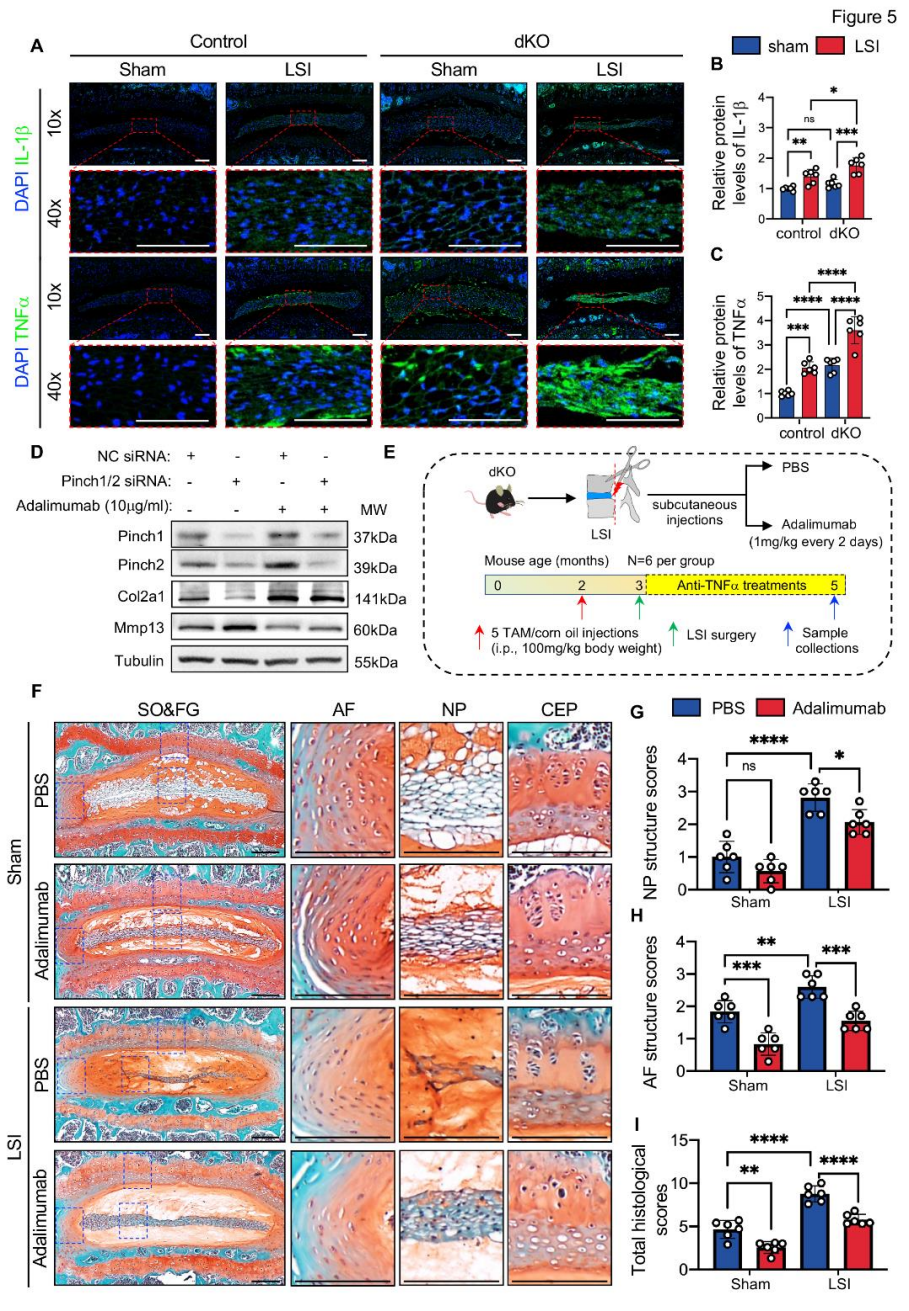
Pharmacological inhibition of TNFα attenuated the DDD-like lesions caused by Pinch loss. (A) IF staining of IL-1β and TNFα in lumbar IVDs of control and dKO mice at 2 months after LSI surgery. The higher magnification images of NP (red dashed boxes) are shown in the lower panels. Scale bar: 200 µm. (B, C) Relative protein levels of IL-1β and TNFα in NP cells normalized to the mean value of the sham-control group. *N = 6* per group. (D) Western blotting analyses of Pinch1, Pinch2, Col2a1, and Mmp13 in NP cells that were transfected with NC siRNA or Pinch1/2 siRNA and treated with or without 10mg/ml Adalimumab. Experiments were repeated three times independently. (E) Overview of Adalimumab treatment experiment. Two-month-old male dKO mice were treated with TAM or corn oil. One week later, the mice were subjected to LSI or sham surgery and then treated with Adalimumab (1mg/kg body weight every 2 days) or PBS for another 2 months. *N = 6* per group. (F) Representative images of SO&FG-stained lumbar IVD sections from PBS- or Adalimumab-treated dKO mice. Blue dashed boxes indicate the higher magnification images of AF, NP, and CEP in the right panels. Scale bar: 200 µm. (G) NP structure scores. (H) AF structure scores. (I) The sum of histological scores. Results are expressed as mean ± standard deviation (s.d.). *P < 0.05; **P < 0.01; ***P < 0.001; ****P < 0.0001; ns: not significant. In B-C and G-I, a two-way ANOVA test was used for statistical analyses.

Results from our and other groups have shown that inflammation plays a crucial role in the initiation and progression of DDD [10, 41, 42]. Thus, we determined whether Pinch loss affects the expression of interleukin-1β (IL-1β) and tumor necrosis factor α (TNFα), both critical pro-inflammatory cytokines, in IVDs of dKO mice with or without LSI surgery. Our results showed that Pinch loss significantly increased the protein expressions of IL-1β and TNFα in LSI-treated dKO IVDs compared to those in LSI-treated control IVDs (Fig. 5A-C). More importantly, we found that the expression of TNFα was increased by 2.16-fold in sham-dKO IVDs versus sham-control IVDs (Fig. 5A, C), suggesting that TNFα might play a vital role in the spontaneous DDD-like lesions caused by Pinch loss. To determine if this was the case, we investigated whether the lesions induced by Pinch loss can be reversed by Adalimumab, a well-known anti-TNFα monoclonal antibody. The results showed that Adalimumab treatment (10 mg/ml) significantly blocked the ECM degradation caused by Pinch knockdown in NP cells in vitro (Fig. 5D). We next investigated whether Adalimumab treatment limits Pinch loss-induced DDD-like lesions in mice. In this experiment, 2-month-old male *Aggrecan^CreERT2^; Pinch1^fl/fl^; Pinch2^-/-^* mice were treated with TAM/corn oil and subjected to LSI/sham surgery, and then treated with PBS or Adalimumab (1mg/kg body weight every 2 days), as indicated in Figure 5E. All mice were killed two months after LSI surgery, and lumbar IVDs were harvested. In control mice, Adalimumab treatment significantly lowered the LSI-induced increases in NP structure scores, AF structure scores, and total histological scores when compared with the PBS treatment group (Supplementary Fig. 9). In dKO mice, Adalimumab treatment remarkably reversed the DDD-like damages caused by Pinch loss with or without LSI (Fig. 5F). Furthermore, Adalimumab treatment markedly reduced the NP structure scores, AF structure scores, NP clefts/fissures scores, and total histological scores in dKO mice with and without LSI (Fig. 5F-I and Supplementary Fig. 10).

**Figure 6.**
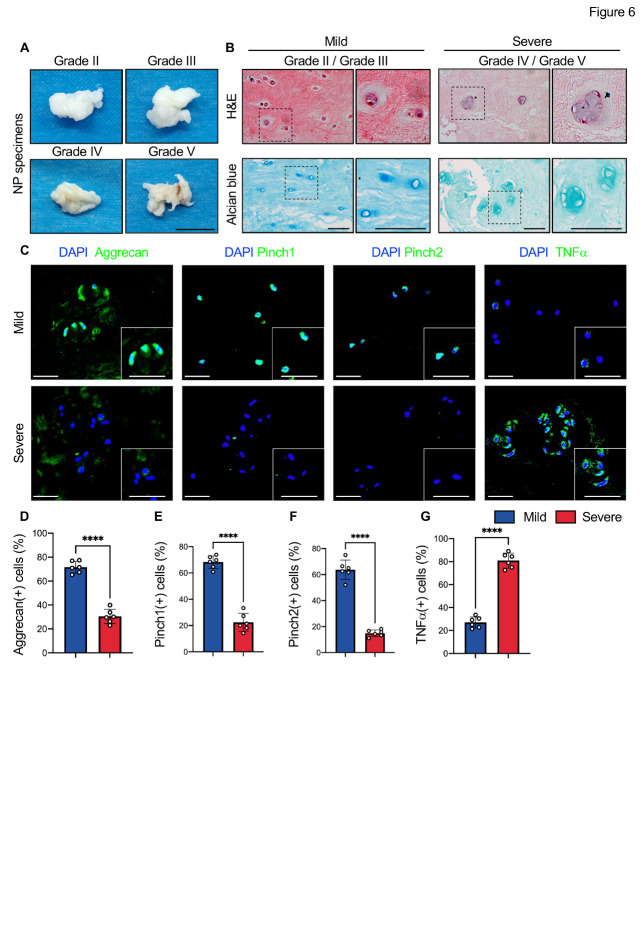
Reduced expression of Pinch proteins in degenerative human NP cells. (A) Representative images of NP specimens isolated from DDD patients with different Pfirrmann degrees. Scale bar, 1 cm. (B) H&E (upper panels) and Alcian blue (lower panels) staining of human NP specimens. Scale bar, 50 μm. (C) IF staining of aggrecan, Pinch1, Pinch2, and TNFα in NP sections from mild or severe DDD patients. The higher magnification images are shown in the right panels. Scale bar: 50 µm. (D-G) Quantitative data of IF staining. *N = 6* biological replications per group. Results are expressed as mean ± standard deviation (s.d.). ****P < 0.0001. In D-G, a two-tailed unpaired Student’s *t* test was used for statistical analyses.

### Reduced Pinch expression in human degenerative NP cells

To investigate whether Pinch expression is altered in human DDD, 12 NP specimens were harvested from DDD patients. The severity of DDD was evaluated from Grade I to Grade IV utilizing a Pfirrmann grading system, and NP specimens with degenerative defects of grade II-III were classified into a mild DDD group, whereas specimens with degenerative defects of grade IV-V were classified into a severe DDD group (*N = 6* per group) (Fig. 6A). H&E stainings revealed several advanced DDD damages, including decreased number of NP cells, fissures, and loss of alcian blue-stained proteoglycan in severe NP samples compared to the mild NP samples (Fig. 6B). IF staining confirmed a significant decrease in the expression of aggrecan in NP sections from the severe group versus the mild group (Fig. 6C, D). Moreover, the percentages of both Pinch1- and Pinch2-positive cells were dramatically reduced, while that of TNFα-positive cells was significantly increased in NP cells from the severe versus mild group (Fig. 6C, E-G).

## DISCUSSION

DDD is a complex disease in which genetic susceptibility interacts with environmental risk factors, leading to irreversible damage to IVD tissues [43]. A degenerated IVD can be characterized by decreased disc height, NP fibrosis and dehydration, buckling of AF lamellae, destruction of CEP, and subchondral bone sclerosis [44]. Although the complicated mechanisms of DDD pathogenesis remain elusive, several key molecules and signaling pathways have been linked to the initiation and progression of DDD, which include but are not limited to Wnt/β-catenin, TGF-β/Smads, TNFα/NFκB, NLRP3/IL-1β, hypoxia-induced factors (HIFs), fibroblast growth factor (FGF), and FA signaling pathways [10, 42, 45-52]. For instance, conditional activation of β-catenin in Col2a1-expressing IVD cells caused degenerative disc defects, such as reduced length of the spine, loss of CEP, and extensive osteophyte formation [46]. Aberrant activation of Hif1α promoted IVD degeneration by enhancing glycolytic metabolism and disrupting mitochondrial functions, whereas Hif1α inhibition decelerated the DDD progression in mice [48]. Results from our group have demonstrated that Kindlin-2, another key FA protein, plays a vital role in maintaining IVD homeostasis by suppressing Nlrp3 activation in mice [10, 53]. In this study, we provide a piece of new knowledge in this field by demonstrating that Pinch1 and Pinch2 acted synergistically to maintain IVD homeostasis in mice. We provide convincing evidence that loss of Pinch1/2 induced progressive DDD-like phenotypes and exacerbated instability-induced disc defects in adult mice. We find that Pinch loss caused spontaneous ECM degradation, cell apoptosis, and inflammation in IVDs. Notably, this is the first demonstration of the pivotal role of Pinch proteins in maintaining IVD homeostasis to protect against the initiation and progression of DDD.

Aging is a major etiologic factor contributing to DDD development and progression [54]. It is known that aging causes degenerative defects in all IVD tissues, including AF, NP, and CEP [55]; however, the molecular mechanism of how aging initiates pathological changes in these areas remains incompletely understood. Results from this study show that Pinch1 and Pinch2 were both highly expressed in cells of AF, NP, and CEP of healthy adult IVDs and dramatically downregulated in aged IVDs. Interestingly, we find that genetic deletion of Pinch proteins in these IVD tissues resulted in multiple striking spontaneous DDD-like defects, including decreased cellularity, NP fibrosis, and loss of boundary between AF and NP, which highly mimic the major pathological features of aging-induced IVD defects. These findings, along with the previous observation that loss of FA-related proteins induced/aggravated aging-related pathological changes [9, 10, 16, 19], suggest a pivotal role of the FA signaling pathway in preserving the IVDs from aging-related degenerative defects.

It should be noted that the compensation between Pinch1 and Pinch2 has also been reported in organs and tissues other than IVDs. For instance, Stanchi et al. have shown that Pinch2 loss caused no apparent abnormalities but a marked upregulation of Pinch1 in the bladder and kidney of Pinch2^-/-^ mice [26]. Shi and coworkers have demonstrated that Pinch2 can substitute for Pinch1 to mediate specific cellular processes, such as cell spreading, survival, and ECM deposition [56]. In addition, results from our groups have reported the functional redundancy of Pinch1 and Pinch2 in regulating skeletal morphogenesis, bone remodeling, glucose metabolism, and adipose tissue mass in mice [29, 30, 32]. In this study, we demonstrate that Pinch1 and Pinch2 expressions compensated for each other in IVDs under physiological conditions; however, this compensation mechanism failed to work under pathological circumstances, such as aging- and LSI-induced DDD. Thus, it seems crucial to keep the Pinch expression at a proper level to protect against DDD development. The mechanisms through which pathological factors, such as aging, inflammation, and mechanical stress, downregulate Pinch expression need to be determined in future studies.

Emerging evidence highlights the critical role of inflammation in contributing to the onset and development of DDD [57, 58]. We recently demonstrated that the deficiency of FA protein Kindlin-2 led to DDD progression through activating the Nlrp3/IL-1 inflammatory pathway [10]. Results from this study suggest that Pinch loss caused DDD-like defects through, at least in part, inducing IVD inflammation. We provide several lines of evidence to support this notion. First, TNFα and IL-1β, the two most potent pro-inflammatory cytokines, were significantly upregulated in IVDs of dKO mice after LSI surgery. Second, compared to control IVDs, Pinch loss dramatically increased the expression of TNFα by 2.16-fold in dKO IVDs without LSI. Third and most importantly, pharmacological inhibition of TNFα largely mitigated the spontaneous and LSI-induced degenerative lesions caused by Pinch loss in dKO mice. Consistent with results from this study, Wang et al. have demonstrated that genetic ablation of Pinch1 in cardiomyocytes activated the NFκB signaling pathway, resulting in excessive production of TNFα and cardiac injury [59]. It has been demonstrated that TNFα promotes DDD progression by causing ECM degradation, amplifying the inflammatory responses, and inducing IVD cell apoptosis [51, 60]. Moreover, a recent study showed that genetic deletion of TNF receptor 1 (Tnfr1), the membrane receptor of TNFα, decelerated the DDD progression, whereas deletion of TNF receptor 2 (Tnfr2) exerted opposite effects [42]. Although anti-TNFα therapies display beneficial effects on DDD-related clinical symptoms, such as low back/neck pain; however, only a few pieces of evidence have been obtained regarding whether and how anti-TNFα therapies can prevent or delay the occurrence of pathological changes in IVD tissues during DDD development. For instance, Evashwick-Rogler and colleagues reported that inhibition of TNFα limited the long-term pain and degenerative disc defects in a rat model of IVD injury [61]. This study shows that the anti-TNFα monoclonal antibody Adalimumab significantly alleviated the DDD-like pathological changes caused by Pinch loss. These findings, along with our observation that TNFα expression was drastically increased in the severe degenerative human NP samples, suggest the critical role of TNFα-mediated inflammation in the pathogenesis of DDD. Interestingly, we find that Pinch deletion markedly increased the protein expression of phosphorylated p65, a key transcriptional factor in NFκB signaling, in NP cells both in vitro and in vivo. Although the precise mechanism is unclear, these findings indicate that loss of Pinch expression may activate NFκB signaling and thus increase the production of downstream molecule TNFα in NP cells. The TNFα can further boost the activation of NFκB signaling, leading to excessive expression of ECM-degrading enzymes, such as Mmp13 and Adamst5, and ECM degradation. Moreover, we find that Pinch loss significantly downregulated the protein expression of total and phosphorylated Smad2/3, suggesting that Pinch loss has an inhibitory effect on the TGF-β/Smads signaling. The inhibition of TGF-β/Smads signaling may also contribute to the Pinch loss-induced DDD-like phenotypes. The molecular mechanism through which Pinch proteins maintain IVD homeostasis requires further investigation.

Cumulative evidence from our and other groups has shown that loss of FA-related proteins, especially the Kindlin-2 and Pinch, led to suppressed cell proliferation and enhanced cell apoptosis in multiple organs and tissues, such as bone, cartilage, adipose tissues, pancreatic islet, and tumors [9, 15, 16, 18, 28-30, 32, 62]. In line with these findings, results from this study reveal that Pinch loss significantly inhibited proliferative activity and induced apoptosis in IVD cells. Notably, Guo and coworkers have demonstrated that Pinch1 promoted the translocation of Kindlin-2 into mitochondria and facilitated the interaction between Kindlin-2 and pyrroline-5-carboxylate reductase 1 (Pycr1), an essential protein in regulating proline biosynthesis [28]. Loss of Pinch1 or Kindlin-2 caused a decrease in Pycr1 expression and proline synthesis, leading to mitochondrial dysfunction and excessive production of reactive oxygen species (ROS), and thus inhibited cell proliferation and induced apoptosis [15, 28].

It is known that Pinch proteins function through the formation of ILK (integrin-linked kinase)-Pinch-Parvin (IPP) complex [24]. The complex formation stabilizes each component, which otherwise is targeted for degradation [23, 63, 64]. Results from this study demonstrate that deletion of either Pinch1 or Pinch2 did not induce marked abnormalities in IVD, whereas the loss of both factors resulted in severe DDD-like lesions in mice. These findings support the notion that Pinch1 and Pinch2 can compensate for each other to form either ILK-Pinch1-Parvin or ILK-Pinch2-Parvin complex in maintaining IVD homeostasis. Nonetheless, our results from this study do not exclude that Pinch can function in IVD in an IPP-independent manner.

We acknowledge that this study has several limitations. First, although our histological data demonstrated that Pinch loss causes severe DDD-like damages, we did not determine whether Pinch loss affects disc mechanical properties and pain-related behaviors. Second, as corn oil-treated mice were used as controls, we did not determine whether tamoxifen treatment itself has effects on disc structure. Third, the pathological factors responsible for the downregulation of Pinch proteins during aging-induced DDD need to be determined. Fourth, although our results indicate that Pinch maintains IVD homeostasis through inhibiting TNFα-induced inflammation in IVDs, detailed molecular mechanisms require further investigation. Fifth, whether overexpression of Pinch1 and/or Pinch2 in IVD tissues can exert protective effects against DDD progression remains to be investigated in future studies. In conclusion, our study demonstrates the crucial role of Pinch proteins in maintaining IVD homeostasis and defines a potential therapeutic target for DDD.

## Supplementary Materials

The Supplementary data can be found online at: www.aginganddisease.org/EN/10.14336/AD.2023.0212.


