# Supplementary material for: Loss of Pinch Proteins Causes Severe Degenerative Disc Disease-Like Lesions in Mice
Source: Aging Dis. 2023 Oct 1;14(5):1818–33. doi: 10.14336/AD.2023.0212 (PMC10529740; doi:10.14336/AD.2023.0212)
Supplement: Supplementary file 1 [file AD-14-5-1818-s.pdf]

SUPPLEMENTARY DATA

Loss of Pinch Proteins Causes Severe Degenerative Disc Disease-Like Lesions in Mice

Xiaohao Wu[#*], Mingjue Chen[#], Sixiong Lin[#], Sheng Chen[#], Jingliang Gu, Yuchen Wu, Minghao Qu, Weiyuan Gong, Qing Yao, Huiping Li, Xuenong Zou, Di Chen, Guozhi Xiao[*]

© 2022. Wu X et al. Published online at http://www.aginganddisease.org/EN/10.14336/AD.2023.0212

SUPPLEMENTARY DATA

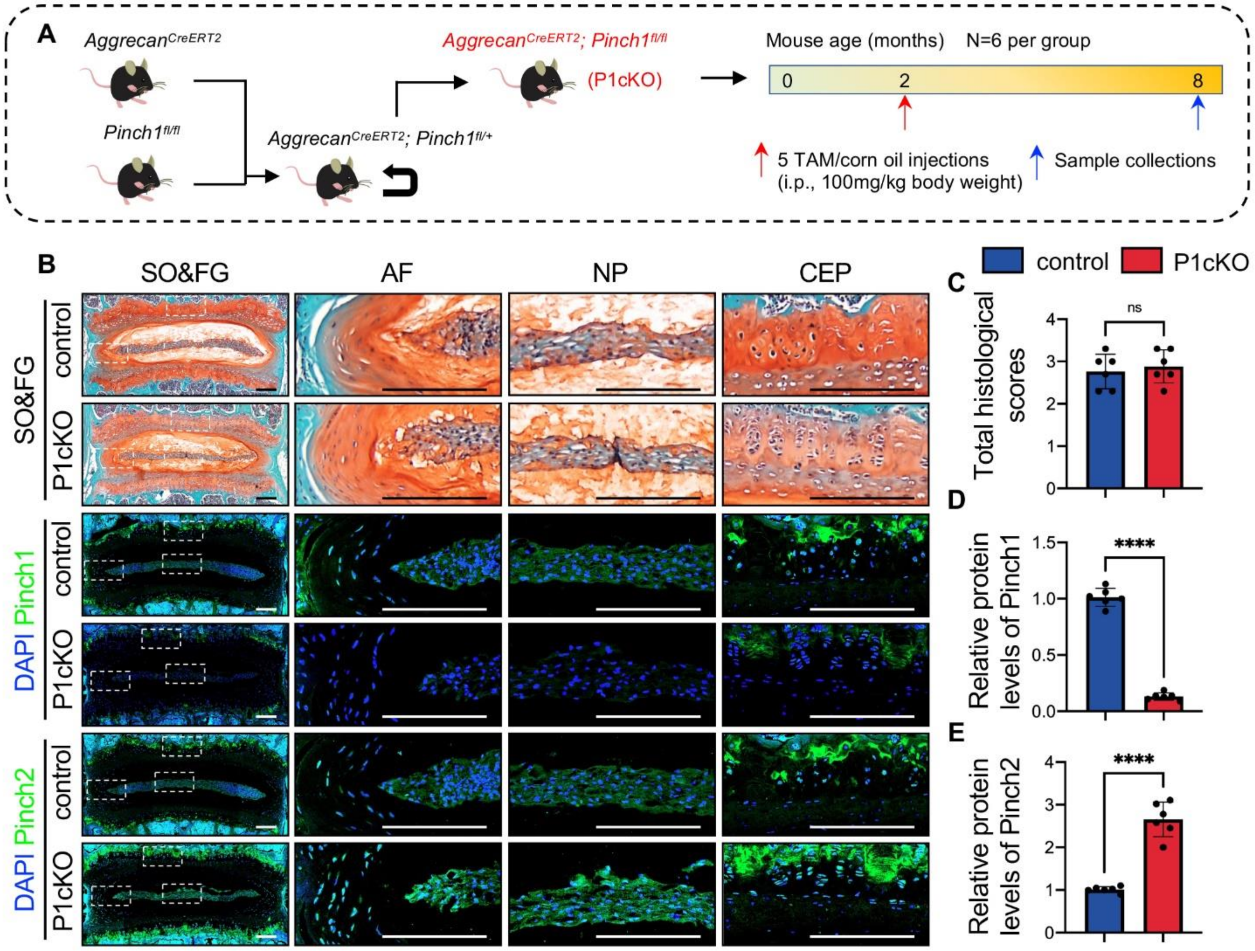

Supplementary Figure 1. Deleting Pinch1 in aggrecan-positive IVD cells did not cause DDD in mice. (**A**) A schematic diagram illustrating the experimental design. *Pinch1*$^{fl/fl}$ mice were bred with *Aggrecan*^{CreERT2} mice to obtain *Aggrecan*^{CreERT2}*; Pinch1*$^{fl/fl}$ mice. At 2 months of age, the *Aggrecan*^{CreERT2}*; Pinch1*$^{fl/fl}$ mice were treated with TAM for inducible deletion of the *Pinch1* gene in aggrecan-expressing IVD cells (P1cKO). The *Aggrecan*^{CreERT2}*; Pinch1*$^{fl/fl}$ mice treated with corn oil were used as controls. $N = 6$ per group. (**B**) Representative images of SO&FG and IF staining of lumbar IVD sections from control and P1cKO mice. White dashed boxes indicate the higher magnification images of AF, NP, and CEP in the right panels. Scale bar: 200 mm. (**C**) Total histological scores of IVDs from control and P1cKO mice at 8 months of age. (**D, E**) Relative protein expression of Pinch1 (D) and Pinch2 (E) in lumbar IVDs, which is normalized to the mean value of the control group. ****$P < 0.0001$; ns: not significant. In C-E, a two-tailed unpaired Student's *t* test was used for statistical analyses.

© 2022. Wu X et al. Published online at http://www.aginganddisease.org/EN/10.14336/AD.2023.0212

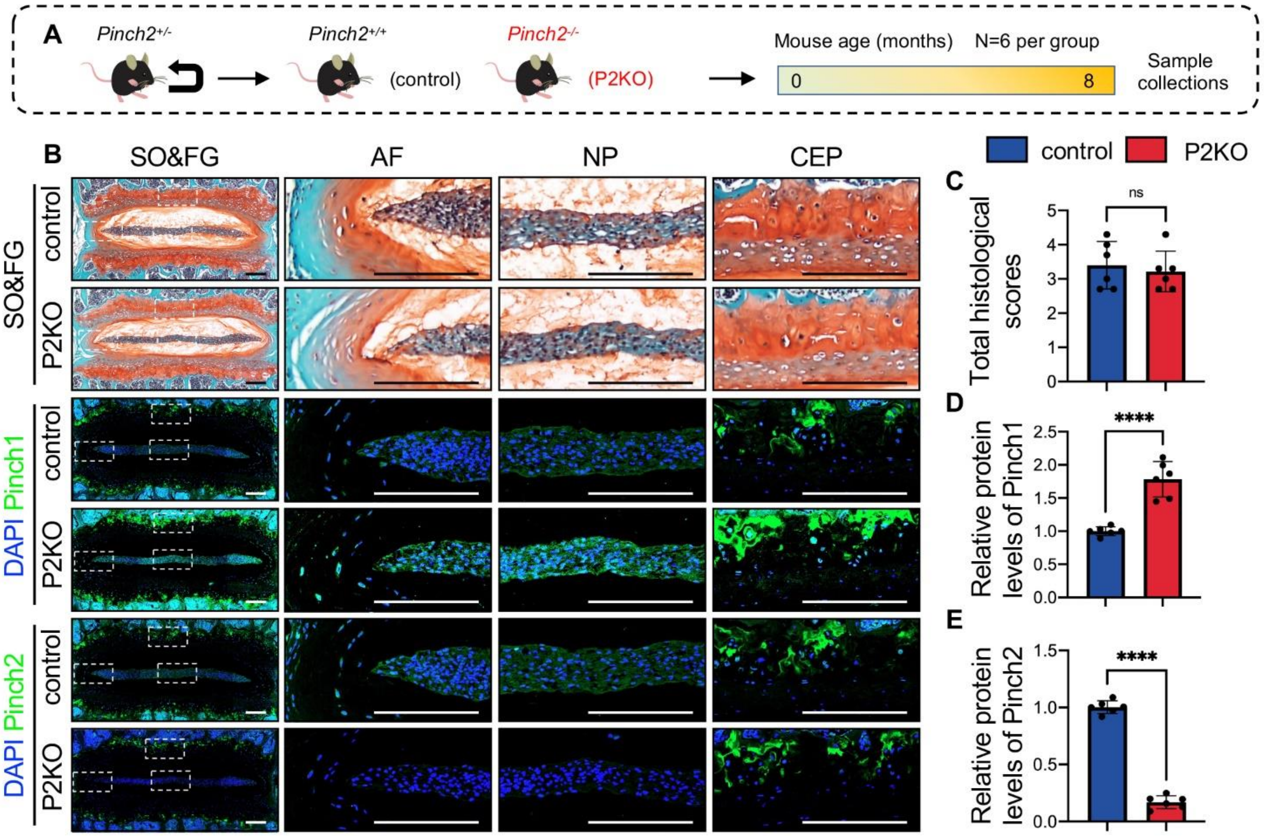

Supplementary Figure 2. Deleting Pinch2 globally did not cause DDD in mice. (**A**) Overview of experiments in Pinch2 global knockout (P2KO) mice. $N = 6$ per group. (**B**) Representative images of SO&FG and IF staining of lumbar IVD sections from control and P2KO mice. White dashed boxes indicate the higher magnification images of AF, NP, and CEP in the right panels. Scale bar: 200 mm. (**C**) Total histological scores of IVDs from control and P2KO mice at 8 months of age. (**D, E**) Relative protein expression of Pinch1 (D) and Pinch2 (E) in lumbar IVDs, which is normalized to the mean value of the control group. ****$P < 0.0001$; ns: not significant. In C-E, a two-tailed unpaired Student's *t* test was used for statistical analyses.

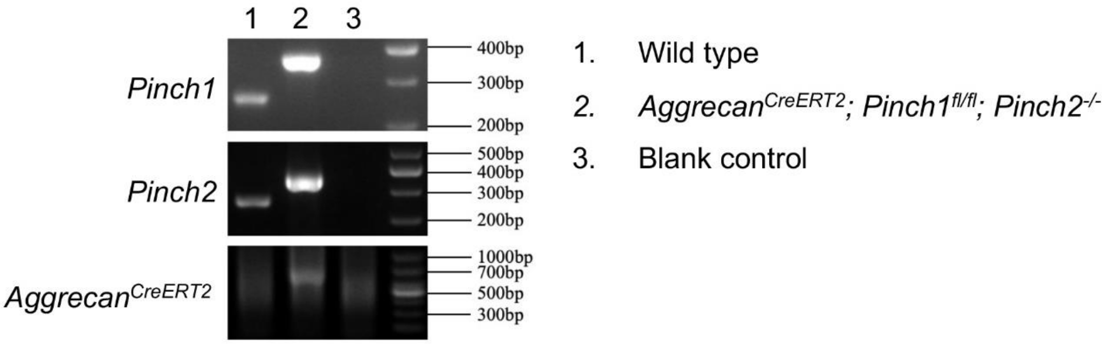

Supplementary Figure 3. Genotyping of *Aggrecan*^{CreERT2}*; Pinch1*$^{fl/fl}$*; Pinch2*$^{-/-}$ mice. PCR genotyping using mouse tail DNA. *Pinch1* flox KO band, ~350bp; *Pinch1* WT band, ~250bp; *Pinch2* KO band, ~350bp; *Pinch2* WT band, ~250bp; *Aggrecan*^{CreERT2}, ~650bp. Primer sets are listed in Supplementary Table 1.

© 2022. Wu X et al. Published online at http://www.aginganddisease.org/EN/10.14336/AD.2023.0212

SUPPLEMENTARY DATA

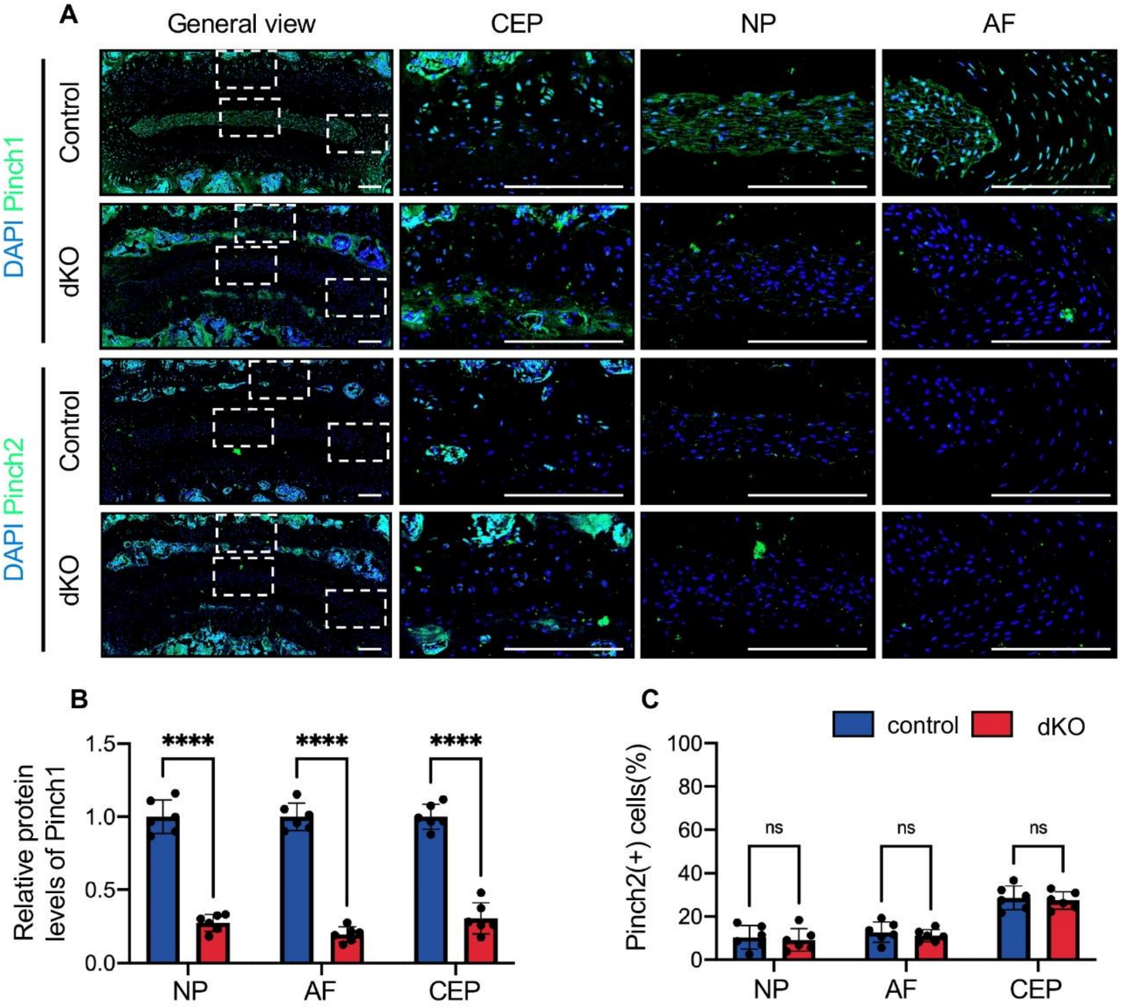

Supplementary Figure 4. Expression of Pinch1 and Pinch2 in lumbar IVDs of control and dKO mice. (**A**) IF staining of Pinch1 and Pinch2 in control or dKO lumbar IVD sections at 5 months after TAM injections. Higher magnification images of CEP, NP, and AF are shown in the right panels. Scale bar: 200 mm. (**B, C**) Quantitative analyses of Pinch1 and Pinch2 expressions in the above areas. Relative Pinch1 levels were calculated by normalizing each measured value to the mean value of the control group. $N = 6$ per group. Results are expressed as mean ± standard deviation (s.d.). $****P < 0.0001$; ns: not significant. In B and C, a two-way ANOVA test was used for statistical analyses.

© 2022. Wu X et al. Published online at http://www.aginganddisease.org/EN/10.14336/AD.2023.0212

SUPPLEMENTARY DATA

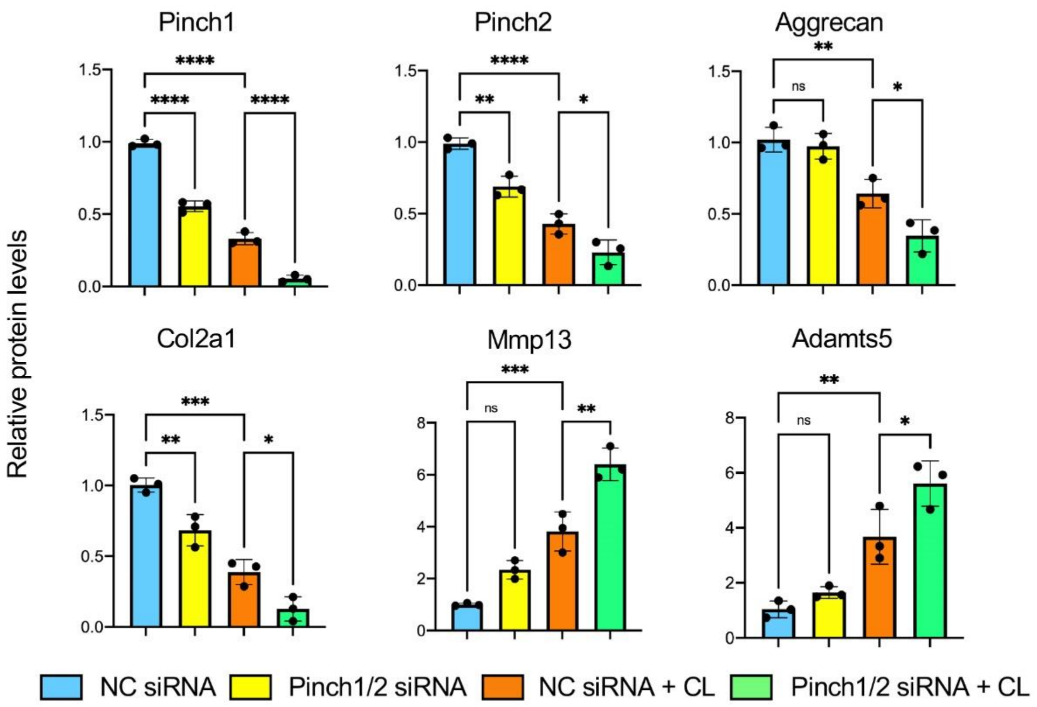

Supplementary Figure 5. Quantitative data of western blotting analyses. The relative protein levels of Pinch1, Pinch2, aggrecan, Col2a1, Mmp13, and Adamts5 in NP cells transfected with negative control siRNA or Pinch1/2 siRNA for 24 hours with or without compression loading (CL) treatment. Experiments were repeated three times independently with similar results. Results are expressed as mean ± standard deviation (s.d.). The relative protein levels were calculated by normalizing each measured value to the mean value of the NC siRNA group. *$P < 0.05$; **$P < 0.01$; ***$P < 0.001$; ****$P < 0.0001$; ns: not significant. A two-way ANOVA test was used for statistical analyses.

© 2022. Wu X et al. Published online at http://www.aginganddisease.org/EN/10.14336/AD.2023.0212

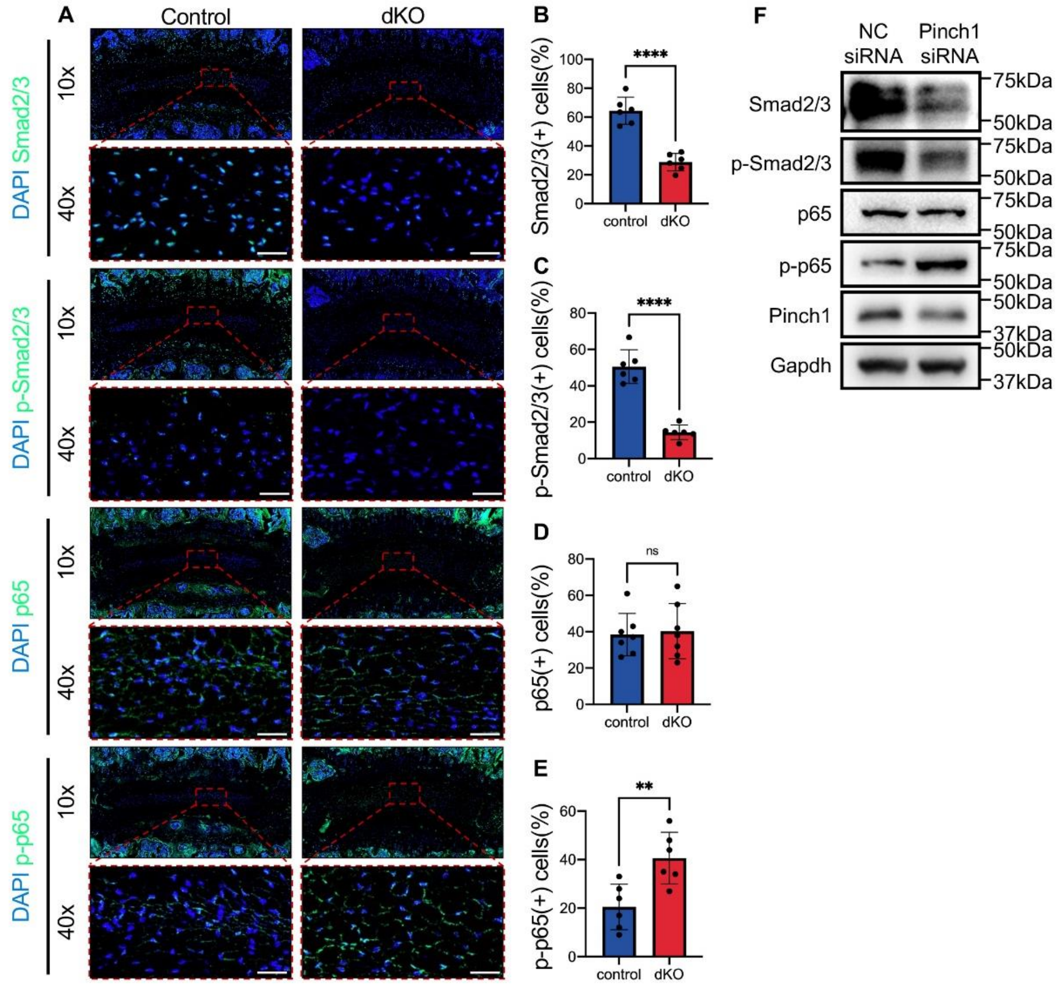

Supplementary Figure 6. Effects of Pinch loss on the activation of TGFb/Smad and NFkB signaling pathways in vivo and in vitro. (**A**) IF staining of Smad2/3, p-Smad2/3, p65, and p-p65 in control or dKO lumbar IVD sections at 13 weeks after TAM injections. Higher magnification images of NP tissue are shown in the lower panels. Scale bar: 200 mm. (**B-E**) Percentages of positively stained cells in NP tissue. $N = 6$ per group. (**F**) Western blotting analyses of protein extracts from NP cells treated with NC siRNA or Pinch1 siRNA for 48 hours. Results are expressed as mean ± standard deviation (s.d.). $^{**}P < 0.01$; $^{****}P < 0.0001$; ns: not significant. In B-E, a two-tailed unpaired Student's *t* test was used for statistical analyses.

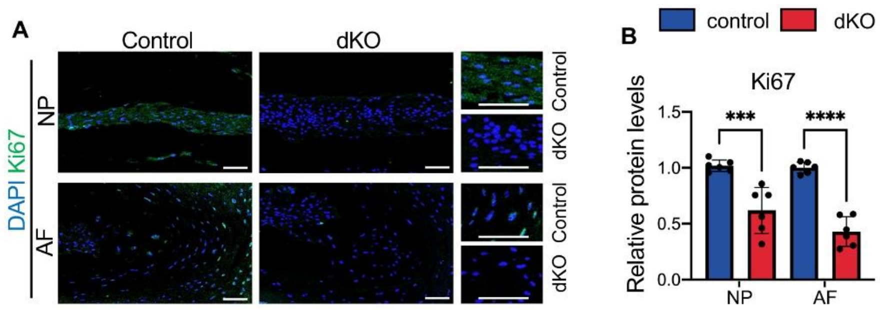

Supplementary Figure 7. Pinch loss inhibited cell proliferation in lumbar IVDs in mice. (**A**) Fluorescent staining of cell proliferating maker Ki67 in control or dKO lumbar IVD sections at 10 months of age. Higher magnification images are shown in the right panels. Scale bar: 200 mm. (**B**) Relative protein expression of Ki67 in NP or AF of lumbar IVDs, which is normalized to the mean value of the control group. $N = 6$ per group. Results are expressed as mean ± standard deviation (s.d.). $^{***}P < 0.001$. $^{****}P < 0.0001$. In B, a two-way ANOVA test was used for statistical analyses.

© 2022. Wu X et al. Published online at http://www.aginganddisease.org/EN/10.14336/AD.2023.0212

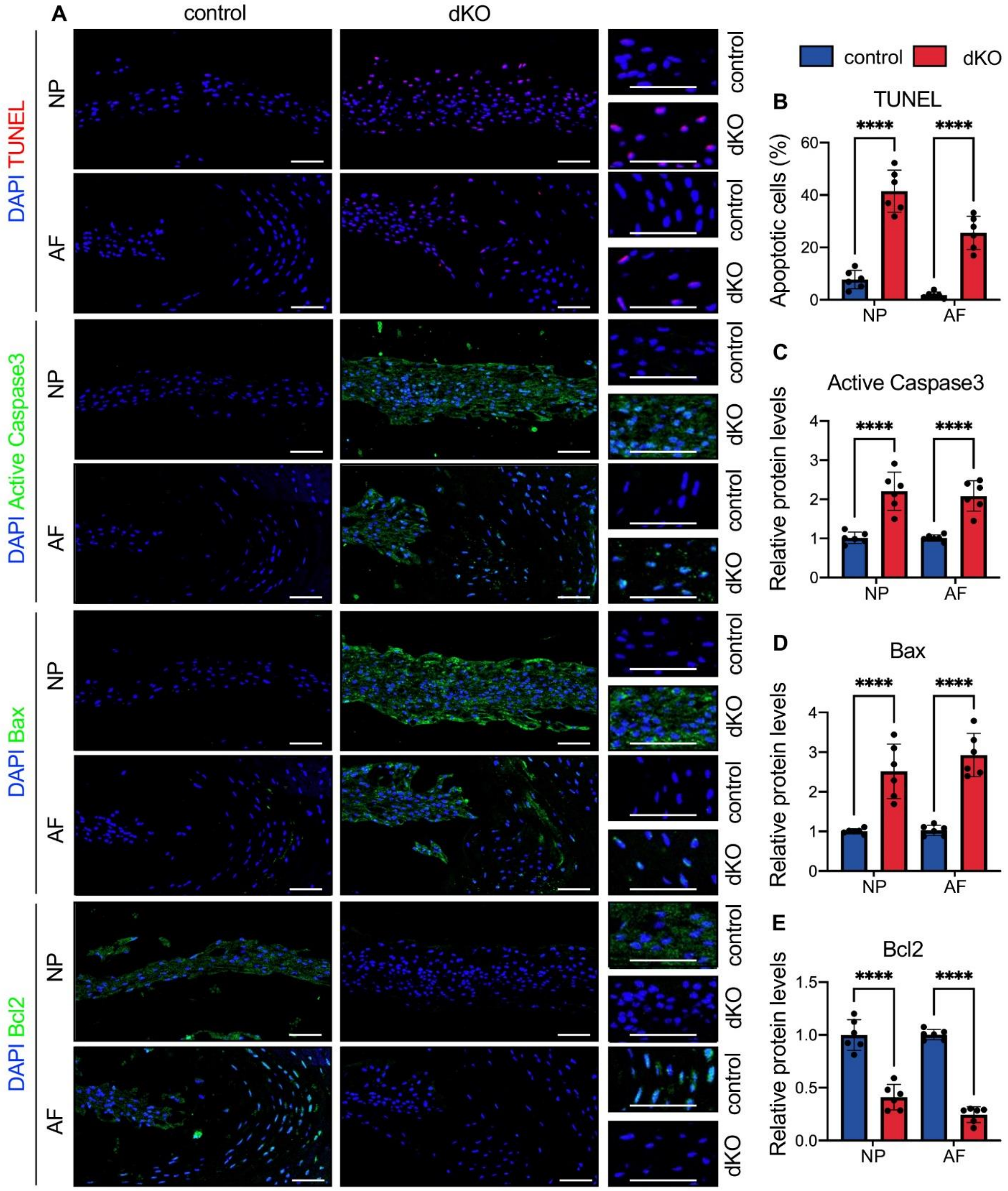

Supplementary Figure 8. Pinch loss promoted cell apoptosis in lumbar IVDs in mice. (**A**) Fluorescent staining of the terminal deoxynucleotidyl transferase-mediated nick end labeling (TUNEL), active Caspase 3, Bax, and Bcl2 in control or dKO lumbar IVD sections at 10 months of age. Higher magnification images are shown in the right panels. Scale bar: 200 mm. (**B**) Percentage of TUNEL-positive apoptotic cells. (**C**-**E**) Relative protein expressions of active Caspase3, Bax, and Bcl2 in NP or AF of lumbar IVDs, which are normalized to the mean value of the control group. $N = 6$ per group. Results are expressed as mean ± standard deviation (s.d.). ****$P <$ 0.0001. In B-E, a two-way ANOVA test was used for statistical analyses.

© 2022. Wu X et al. Published online at http://www.aginganddisease.org/EN/10.14336/AD.2023.0212

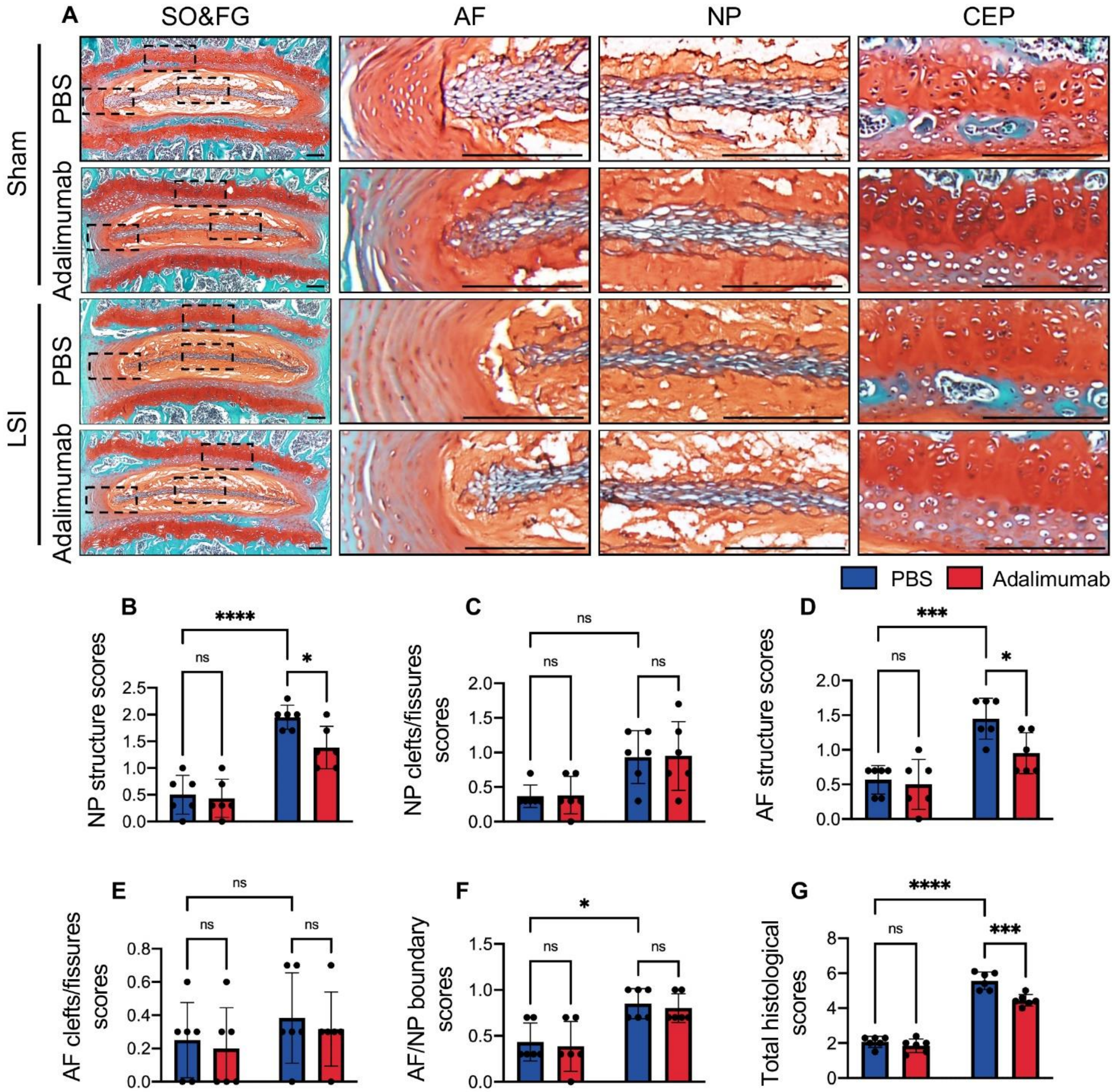

Supplementary Figure 9. Effects of Adalimumab on IVD morphology in control mice. (**A**) Representative images of SO&FG staining of lumbar IVD sections from control mice treated with PBS or Adalimumab at 8 weeks after LSI surgery. Black dashed boxes indicate the higher magnification images of AF, NP, and CEP in the right panels. Scale bar: 200 mm. (**B-G**) Histological scores. $N = 6$ per group. Results are expressed as mean ± standard deviation (s.d.). *$P < 0.05$; ***$P < 0.001$; ****$P < 0.0001$; ns: not significant. In B-G, a two-way ANOVA test was used for statistical analyses.

© 2022. Wu X et al. Published online at http://www.aginganddisease.org/EN/10.14336/AD.2023.0212

SUPPLEMENTARY DATA

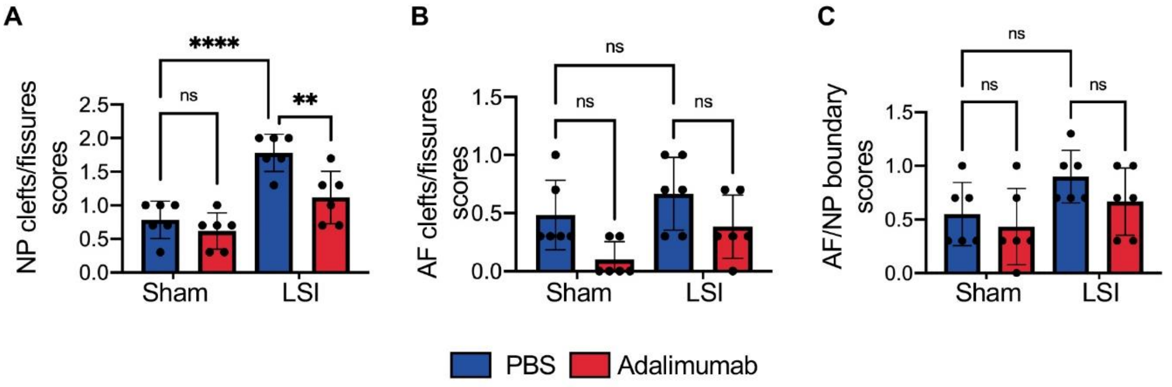

Supplementary Figure 10. Effects of Adalimumab on IVD morphology in dKO mice. (**A**) NP clefts/fissures scores. (**B**) AF clefts/fissures scores. (**C**) AF/NP boundary scores. $N = 6$ per group. Results are expressed as mean ± standard deviation (s.d.). $^{**}P < 0.01$; $^{****}P < 0.0001$; ns: not significant. A two-way ANOVA test was used for statistical analyses.

Supplementary Table 1. Demographic information of the DDD patients enrolled in the study.

Case no.	Age (years)	Sex	Disc level	Pfirrmann grading
1	42	F	L4/5	II
2	56	M	L4/5	II
3	29	F	L5/S1	II
4	35	F	L5/S1	III
5	77	M	L4/5	III
6	53	M	L5/S1	III
7	33	M	L4/5	IV
8	59	M	L5/S1	IV
9	44	F	L4/5	IV
10	67	F	L5/S1	V
11	81	F	L4/5	V
12	59	M	L5/S1	V

Supplementary Table 2. Genotyping primer information

**Gene**	**Forward (5'-3')**	**Reverse (5'-3')**
Pinch1	CCCAGAAGGACTCTTTTATGAG	CTTGGAGAAGAAGTACTCAGGT
Pinch2-p1p2	CTGGAGGGACTGAGCTGGTC	GGGGCCACAGAAGAAAAGAT
Pinch2-p3p4	AATGGGCTGACCGCTTCCTCGT	TGTCATGAGTAATTGGGATTCG
Aggrecan^{CreERT2}	GATCTCCGGTATTGAAACTCCAGC	GCTAAACATGCTTCATCGTCGG

Supplementary Table 3. Antibody information.

**Primary Antibody**	**Company**	**Catalog #**	**Application/Dilution**
Pinch1	Abcam	ab108609	WB (1:1000); IF (1:200)
Pinch2	Abcam	Ab272666	WB (1:1000); IF (1:200)
Aggrecan	ABclonal	A8536	WB (1:1000); IF (1:200)
Col2a1	ABclonal	A1560	WB (1:1000)
Col2a1	Proteintech	28459-1-AP	IF (1:200)
Mmp13	Abcam	ab39012	WB (1:1000); IF (1:200)
Adamts5	Abcam	ab41037	WB (1:1000); IF (1:50)

© 2022. Wu X et al. Published online at http://www.aginganddisease.org/EN/10.14336/AD.2023.0212

Smad2/3	CST	8685S	WB (1:1000); IF (1:200)
p-Smad2/3	CST	8828S	WB (1:1000); IF (1:200)
p65	CST	8242T	WB (1:1000); IF (1:200)
p-p65	CST	3033T	WB (1:1000); IF (1:200)
IL-1β	ABclonal	A1112	WB (1:1000); IF (1:200)
TNF-α	Abcam	ab1793	IF (1:200)
Active caspase 3	Sigma-Aldrich	C8487	WB (1:1000); IF (1:200)
Bcl2	BOSTER	A00040	WB (1:1000); IHC (1:100); IF (1:200)
Bax	CST	2772	WB (1:1000); IHC (1:100); IF (1:200)
Tubulin	CWBIO	CW0098	WB (1:1000)
Isotype antibody (normal rabbit IgG)	Sigma	NI01	IF (1:200)
**Second Antibody**	**Company**	**Catalog #**	**Application/Dilution**
Goat anti-Rabbit IgG (H+L) Cross-Adsorbed Secondary Antibody, Alexa Fluor 488	Invitrogen	A-11008	IF (1:200)

Supplementary Table 4. siRNA sequences

siRNA	Sense (5’-3’)	Antisense (5’-3’)
Pinch1	CCGUGUCAUAGAAGGUGAUTT	AUCACCUUCUAUGACACGGTT
Pinch2	CUCACUCUAAAGAACAAGUTT	ACUUGUUCUUUAGAGUGAGTT

© 2022. Wu X et al. Published online at http://www.aginganddisease.org/EN/10.14336/AD.2023.0212
